# Nuclear Translocation of hARD1 Contributes to Proper Cell Cycle Progression

**DOI:** 10.1371/journal.pone.0105185

**Published:** 2014-08-18

**Authors:** Ji-Hyeon Park, Ji Hae Seo, Hee-Jun Wee, Tam Thuy Lu Vo, Eun Ji Lee, Hoon Choi, Jong-Ho Cha, Bum Ju Ahn, Min Wook Shin, Sung-Jin Bae, Kyu-Won Kim

**Affiliations:** 1 SNU-Harvard NeuroVascular Protection Research Center, College of Pharmacy and Research Institute of Pharmaceutical Sciences, Seoul National University, Seoul, Korea; 2 Department of Molecular Medicine and Biopharmaceutical Sciences, Graduate School of Convergence Science and Technology, and College of Medicine or College of Pharmacy, Seoul National University, Seoul, Korea; Southern Illinois University School of Medicine, United States of America

## Abstract

Arrest defective 1 (ARD1) is an acetyltransferase that is highly conserved across organisms, from yeasts to humans. The high homology and widespread expression of ARD1 across multiple species and tissues signify that it serves a fundamental role in cells. Human ARD1 (hARD1) has been suggested to be involved in diverse biological processes, and its role in cell proliferation and cancer development has been recently drawing attention. However, the subcellular localization of ARD1 and its relevance to cellular function remain largely unknown. Here, we have demonstrated that hARD1 is imported to the nuclei of proliferating cells, especially during S phase. Nuclear localization signal (NLS)-deleted hARD1 (hARD1ΔN), which can no longer access the nucleus, resulted in cell morphology changes and cellular growth impairment. Notably, hARD1ΔN-expressing cells showed alterations in the cell cycle and the expression levels of cell cycle regulators compared to hARD1 wild-type cells. Furthermore, these effects were rescued when the nuclear import of hARD1 was restored by exogenous NLS. Our results show that hARD1 nuclear translocation mediated by NLS is required for cell cycle progression, thereby contributing to proper cell proliferation.

## Introduction

Cell cycle progression is a highly ordered set of events, in which a variety of regulatory proteins work cooperatively. The cell cycle has several checkpoints to prevent inappropriate division of damaged cells, consequently helping to maintain genomic stability. Most cancer cells have mutations in genes that regulate cell cycle checkpoints, leading to uncontrolled proliferation. Therefore, cell cycle regulation is important for the development of anticancer therapies.

The acetyltransferase, arrest defective 1 (ARD1), was initially identified in yeast as a mating-type switch that controls the mitotic cell cycle and alternative development [1], [2]. Further studies have shown that ARD1 is present in various species, including the mouse, rat, chimpanzee, and human, and has several variants, playing different roles [3]–[6]. In humans, human ARD1^235^ (hARD1) is the major form, involved in diverse biological processes, such as cell proliferation, differentiation, autophagy, and cancer [7]–[13].

Recent studies have suggested hARD1 to be oncogenic. Overexpression of hARD1 increased cell proliferation, whereas hARD1 silencing inhibited cellular growth; in addition, hARD1 is highly expressed in several types of cancers, including breast, prostate, lung, and colorectal [12]–[16]. However, conflicting results demonstrate that hARD1 is tumor suppressive, making it complicated to understand the functional consequences of the protein in cancer [11], [17].

The subcellular localization of ARD1 has been previously described by several groups [18]–[20]. Though the presence of a putative nuclear localization signal (NLS) suggests that ARD1 might be localized to the nucleus, some studies have raised questions about its actual validity [18], [19]. Earlier observations on the subcellular localization of ARD1 are also ambiguous and conflicting. Arnesen et al. demonstrated that the majority of hARD1 is present in the nucleus, with low expression in the cytoplasm, in HeLa, GaMg, HEK293, and MCF-7 cells [18]. In contrast, predominant cytoplasmic localization has been observed in HeLa and LoVo cells by other groups [19]. In light of these discrepant results, Kuo et al. suggested distinct localization of hARD1 in different cell lines [7]. It was also suggested that different isoforms of ARD1 (hARD1, mouse ARD1 (mARD1^225^, mARD1^235^)) have different cellular distribution, showing the complicated properties of subcellular localization of ARD1 [3]. On the other hand, another report proposed that the N-terminal region of ARD1 (a.a. 1–35) is responsible for its nuclear localization [20].

Therefore, the subcellular location of ARD1 and its relevance to cellular function warrant more detailed investigation. In the current study, we found that hARD1 nuclear translocation was mediated by its functional NLS, and this translocation helps proper cell cycle progression, consequently contributing to cellular growth.

## Results

### hARD1 is imported to the nucleus during the S phase

**Figure 1:**
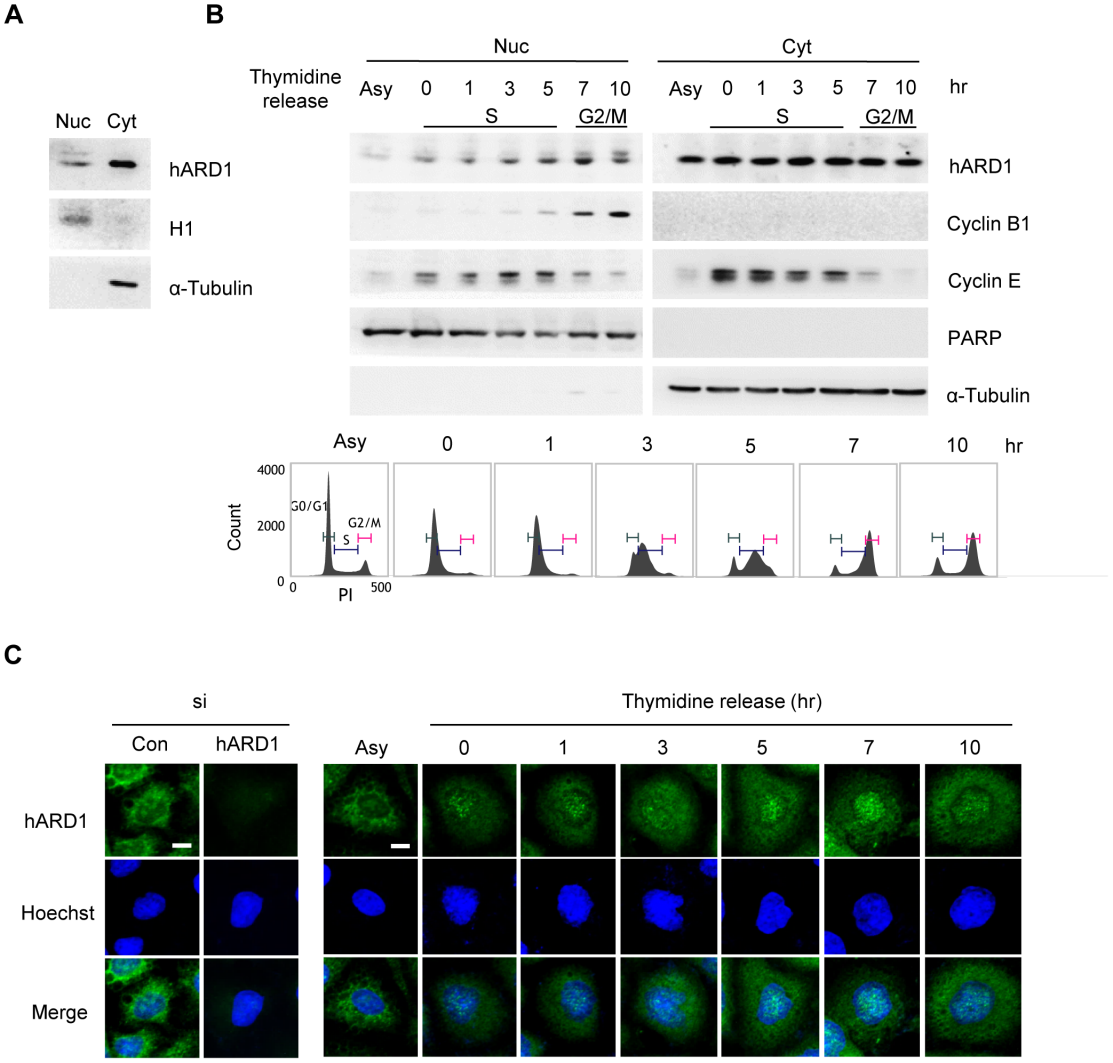
Nuclear hARD1 levels increase during the S phase. **A**. hARD1 predominantly localizes to the cytoplasm of HeLa cells. HeLa cell lysate was subjected to nuclear/cytosolic fractionation, and hARD1 in each compartment was analyzed by immunoblotting. Histone H1 and α-tubulin were included as nuclear and cytoplasmic markers, respectively. Nuc, nuclear protein; Cyt, cytosolic protein. **B** and **C**. hARD1 translocates to the nucleus during S phase. B, HeLa cells were synchronized at the early S phase by double thymidine block, which was released for the indicated times, and then hARD1 subcellular localization was analyzed by performing western blot. Approximations of the cell cycle stages were based on the level of cyclins and were confirmed using flow cytometry. PARP and α-tubulin were included as nuclear and cytoplasmic markers, respectively. Asy, asynchronized cells. C, hARD1 subcellular localization was assessed by immunofluorescence staining. The nuclei were labeled with Hoechst (blue). Scale bar, 10 µm.

To investigate the cellular distribution of hARD1, we conducted nuclear/cytosolic fractionation of HeLa cells and then performed blotting for hARD1. Similar to the findings in several earlier reports [19], hARD1 was predominantly present in the cytoplasm with low levels in the nucleus (Fig. 1A). Because the function of hARD1 has mainly been correlated to cell growth, we investigated whether its localization changed when cell proliferation occurred. HeLa cells were serum-starved for 48 h and re-stimulated with 10% serum, and hARD1 localization was monitored at each indicated time (Fig. S1). Nuclear hARD1 levels were initially low, just after serum starvation, but they increased over time, whereas cytosolic hARD1 showed no change. A decrease in cyclin E, a manager of the G1/S transition, suggested that the cells, which were initially synchronized in G1 by serum starvation, passed through the S phase and finally reached the G2 phase 24 h after serum re-stimulation. To assess the precise time points of hARD1 entry into the nucleus, we synchronized cells to the early S phase with a double thymidine block and collected cells over short time intervals. hARD1 translocated to the nucleus during the S phase and reached its peak around 7 h after release, when the elevated cyclin E levels started to decrease again, potentially in the G2 phase (Fig. 1B, C).

### hARD1 has a functional nuclear localization signal

**Figure 2:**
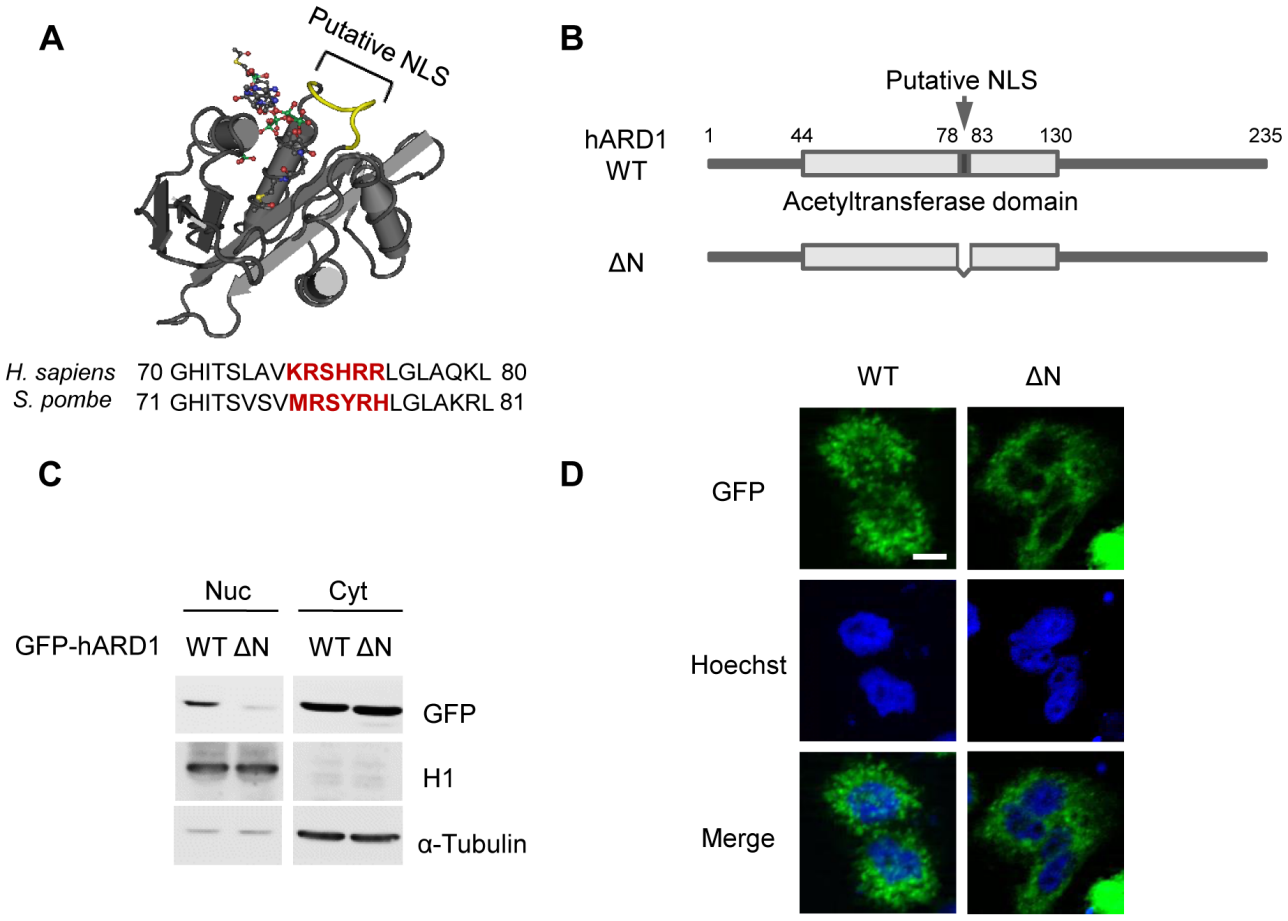
The deletion of the putative NLS of hARD1 blocks nuclear localization. **A**. The structure and sequence comparison of the putative NLS in ARD1. The reported structure of yeast ARD1 is shown. The region corresponding to the NLS in hARD1 (a.a. 79–84, MRSYRH in yeast [21]) is colored yellow. **B**. Schematic presentation of an NLS deletion mutant of hARD1. Wild type hARD1 was tagged with GFP at its N-terminus (GFP-hARD1 WT), and the putative NLS was deleted (GFP-hARD1ΔN). **C**. Deletion of NLS compromised nuclear translocation of hARD1. HEK293T cells transfected with GFP-hARD1 WT and ΔN were subjected to nuclear/cytosolic fractionation and GFP-hARD1 levels were detected by anti-GFP antibody. Note the slight decrease in size of ΔN relative to WT. **D**. NLS-deleted hARD1 was unable to access the nucleus. GFP-hARD1 WT- and ΔN-expressing HeLa cells were visualized by fluorescence microscopy. Scale bar, 10 µm.

The observed changes in nuclear hARD1 suggested nuclear control by a functional NLS. As previously reported [18], hARD1 contains a putative NLS between a.a. 78–83 (KRSHRR), which corresponds to a.a. 79–84 (MRSYRH) of its yeast ortholog [21] (Fig. 2A).

To verify that the putative NLS is a valid, functional NLS, we generated GFP-fused hARD1 wild type (WT) and NLS deletion (ΔN) constructs (Fig. 2B). Upon transient transfection in HEK293T cells for verifying the expression, hARD1 WT was found in both the cytoplasm and nucleus, whereas its deletion mutant localized almost exclusively to the cytoplasm, with little or no presence in the nucleus (Fig. 2C). This was also observed by fluorescence microscopy in stably transfected HeLa cells (Fig. 2D), suggesting that the putative NLS motif is actually required for the nuclear localization of hARD1.

### NLS deletion from hARD1 impairs cell growth

**Figure 3:**
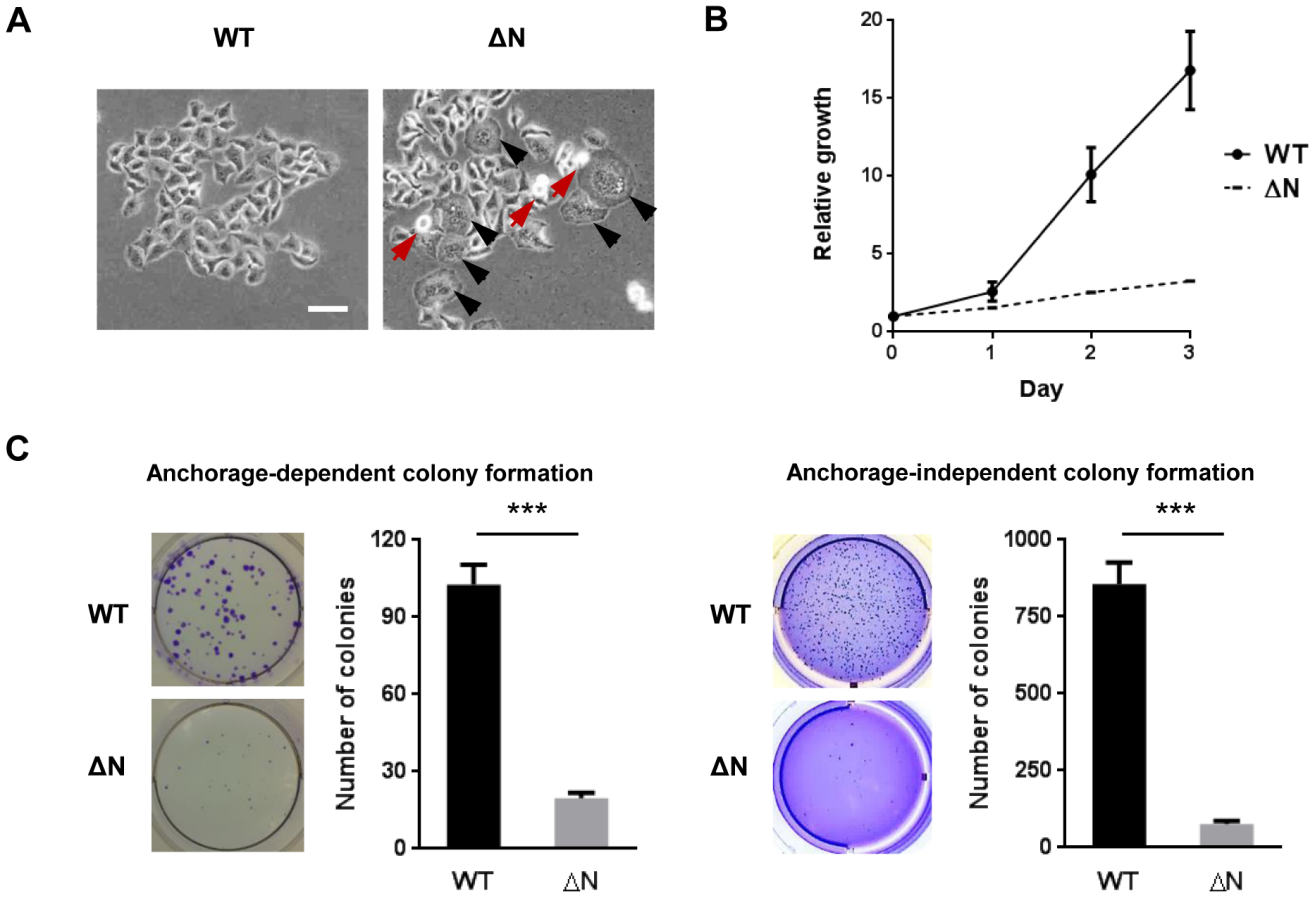
The hARD1 NLS mutant suppresses cell proliferation. **A**. Constitutive expression of the NLS mutant of hARD1 leads to morphological alterations. HeLa cells transfected with hARD1 WT and ΔN were cultured in DMEM containing G418 to select for stable clones. After establishment, cell images were observed by phase-contrast microscopy. Arrows indicate cells with altered morphology (black, enlarged; red, rounded). Scale bar, 50 µm. **B** and **C**. hARD1ΔN-expressing cells showed a marked decrease in cell growth. B, cell growth of hARD1 WT and ΔN stable cell lines was monitored over time by MTS assay. The results are normalized to 0 day of each group and presented as mean ± S.D. (n = 5). C, HeLa cells stably expressing hARD1 WT and ΔN were subjected to anchorage-dependent (left) and -independent (right) colony formation assays. Number of colonies were presented as mean ± S.D. with representative well pictures. N = 3, ***P<0.005.

The nuclear import of hARD1 during cell cycle progression implicates a role in cell proliferation. To determine the effect of hARD1 nuclear translocation on cell growth, we generated HeLa cell lines that stably expressed GFP-hARD1 WT or the ΔN mutant (Fig. 2D). Interestingly, by phase-contrast microscopy, hARD1ΔN mutant-expressing cells showed a distinct morphology - an irregular population of rounded and enlarged cells - compared to WT cells (Fig. 3A).

Moreover, we observed a marked decrease in cell growth when hARD1ΔN was constitutively expressed (Fig. 3B). hARD1ΔN-expressing cells also formed fewer colonies than WT-expressing cells in anchorage-dependent and anchorage-independent colony formation assays (Fig. 3C). These results indicate that nuclear translocation of hARD1 is important in cell growth.

### NLS deletion from hARD1 causes alterations in cell cycle

**Figure 4:**
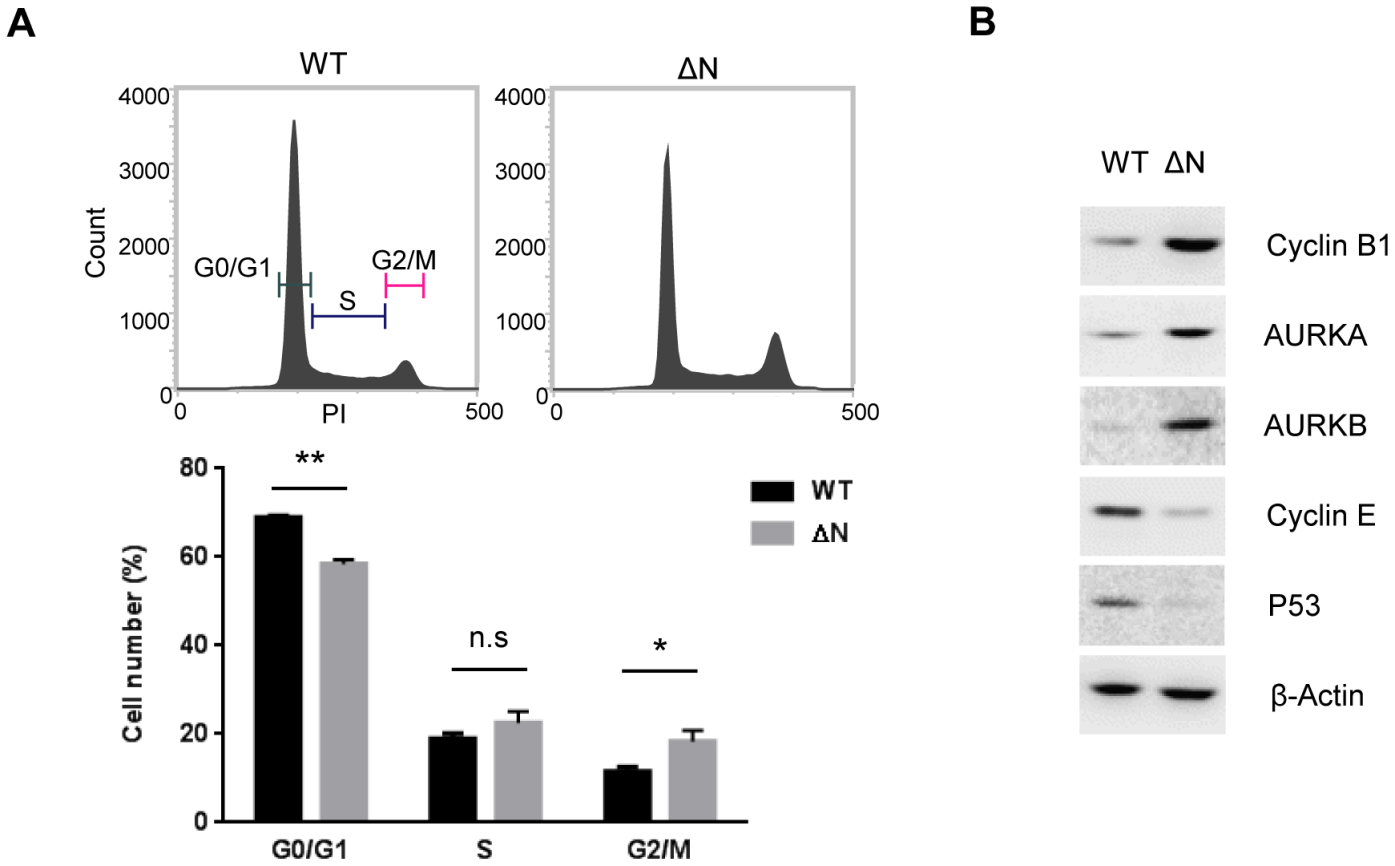
The hARD1 NLS mutant causes alterations in cell cycle. **A**. Deletion of NLS from hARD1 leads to moderate G2/M arrest in HeLa cells. Cell cycle profiles of hARD1 WT- and ΔN-expressing cells were determined by flow cytometry. Upper, representative cell cycle profiles; lower, the percentage of cells in each phase is presented as mean ± S.D. (n = 3). *p<0.05, **P<0.01 **B**. Deletion of NLS from hARD1 increased the protein levels involved in the G2/M phase and decreased those involved in the G0/G1 phase. The levels of Cyclin B1/E, Aurora kinase A/B (AURKA/B), and p53 were immunoblotted in the lysates extracted from hARD1 WT- and ΔN-expressing HeLa cells.

Since NLS deletion from hARD1 caused impairment in cell proliferation, we examined whether the cell cycle distribution was affected using flow cytometry. The results showed that the overexpression of hARD1ΔN increased the G2/M population and decreased the G0/G1 population, which is quite similar to the mild G2/M arrest (Fig. 4A). The expression of proteins involved in cell cycle progression also paralleled this finding. The G2/M phase regulatory proteins, cyclin B1 and aurora kinase A and B (AURKA/B), are increased in hARD1ΔN-expressing cells compared to WT-expressing cells, whereas cyclin E and p53 are decreased (Fig. 4B). Altogether, these results suggest that the reduced proliferation of hARD1ΔN cells is due to cell cycle alterations, which causes an accumulation of cells in the G2/M phase.

### Insertion of exogenous NLS to hARD1ΔN rescues its nuclear import

**Figure 5:**
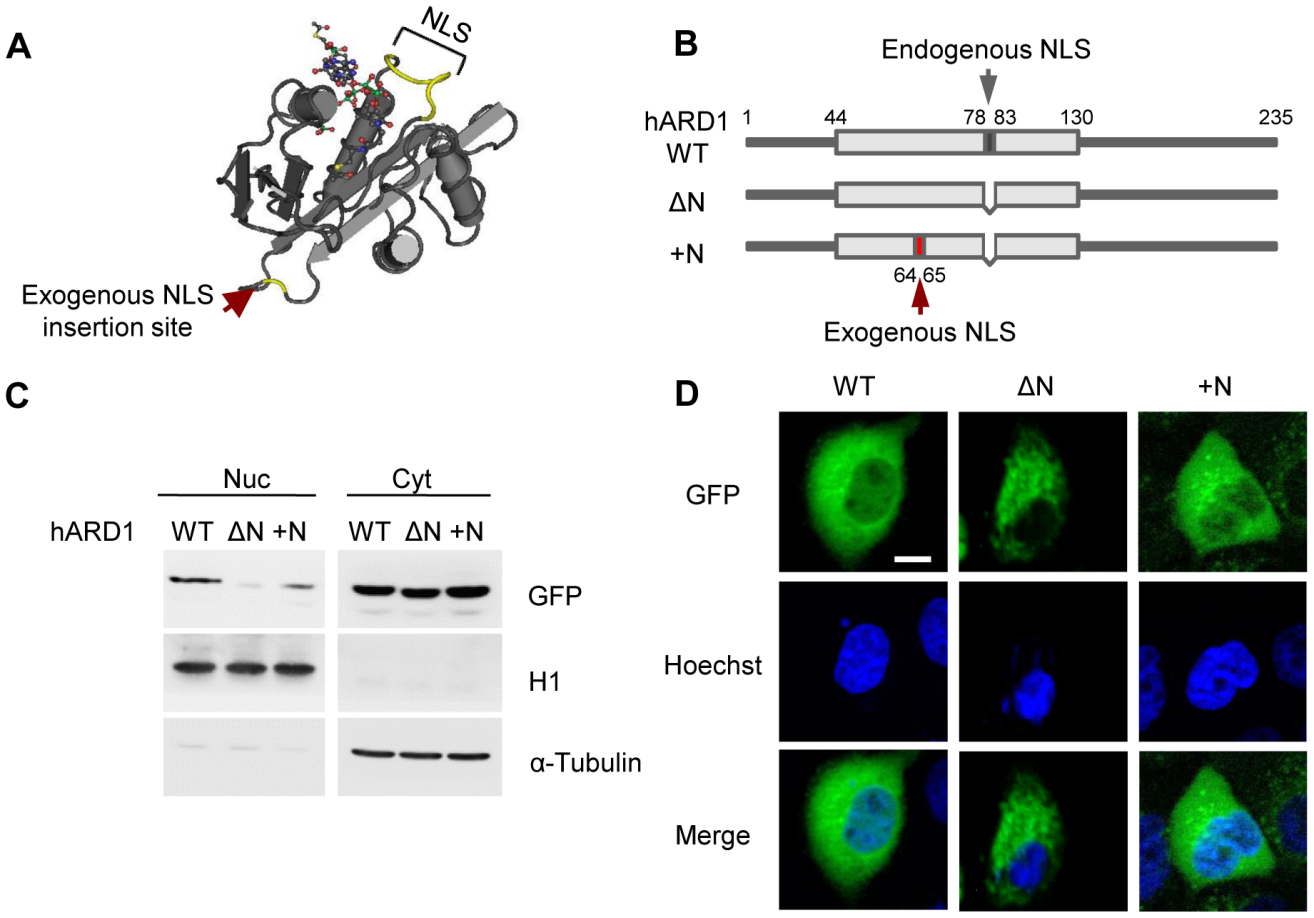
An exogenous NLS can rescue the nuclear localization of the hARD1 NLS mutant. **A**. The region of the exogenous NLS insertion in ARD1. On the reported structure of the yeast ARD1, the region corresponding to the exogenous NLS insertion site in hARD1 (next to a.a. 64) is indicated by a red arrow. **B**. Schematic presentation of an exogenous NLS insertion to the NLS deletion mutant of hARD1. The NLS of hARD1, KRSHRR, was inserted next to a.a. 64 of the NLS deletion mutant (GFP-hARD1+N). **C**. Insertion of NLS into hARD1 rescued the nuclear localization of the NLS deletion mutant. GFP-hARD1 WT, ΔN, and +N were transfected into HEK293T cells, and the localization of hARD1 was analyzed by nuclear/cytosolic fractionation. Note the slightly smaller size of ΔN recovered in +N. **D**. An exogenous NLS can redirect the hARD1 NLS mutant into the nuclei. GFP-hARD1 WT-, ΔN-, and +N-expressing HeLa cells were visualized under fluorescence microscopy. Scale bar, 10 µm.

Though interesting, there is also a chance that deletion of NLS could cause structural changes and consequent aggregation of hARD1 proteins, which might result in random damage to cells. To exclude this possibility, we inserted the exogenous NLS into the hARD1ΔN mutant (hARD1+N) to artificially target it to the nucleus. To minimize the functional alterations caused by inserted residues, we added exogenous NLS into the loop nearest to its original position and not to the N- or C-termini because the N-terminal is crucial for interaction with NATH, and the C-terminal is still unstructured (Fig. 5A, B, red arrows) [21].

The insertion of the exogenous NLS successfully delivered the GFP-hARD1 to the nucleus in transiently transfected HEK293T cells (Fig. 5C). This restoration of nuclear localization was also confirmed by fluorescence microscopy in stably transfected HeLa cells (Fig. 5D).

### Restoration of nuclear localization rescued the impaired cell cycle caused by hARD1ΔN

**Figure 6:**
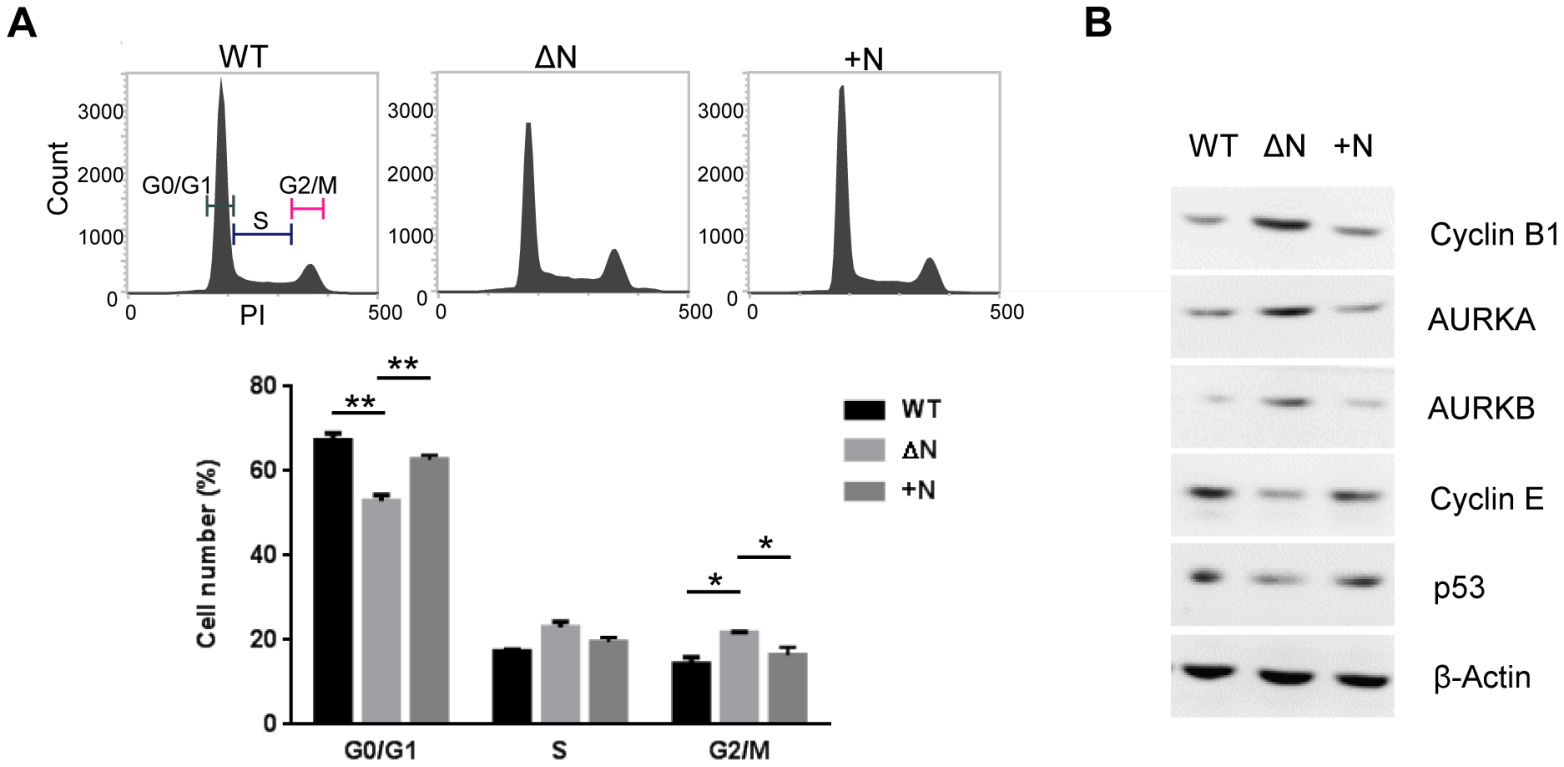
NLS insertion into hARD1 rescues the impaired cell cycle. **A**. Insertion of NLS rescued hARD1ΔN-expressing HeLa cells from G2/M arrest. Cell cycle profiles of stable HeLa cells were determined by flow cytometry analysis. Upper, representative cell cycle profiles; lower, the percentage of cells in each phase. **B**. The exogenous NLS restored the altered protein expression involved in cell cycle regulation of the NLS mutant. The levels of Cyclin B1/E, AURKA/B, and p53 were determined in cell extracts from hARD1 WT-, ΔN-, and +N-expressing cells by western blot.

We next examined whether the restoration of nuclear localization by exogenous NLS could rescue the cell cycle alterations. As observed by flow cytometry, the increased G2/M population in hARD1ΔN-expressing cells was restored to similar levels as WT after the addition of the NLS (Fig. 6A). Moreover, expression levels of proteins involved in regulating cell cycle progression were also restored by exogenous NLS (Fig. 6B), indicating that the altered cell cycle of hARD1ΔN cells was, in fact, caused by abnormal nuclear translocation.

### Restoration of nuclear localization rescued the impaired cell growth caused by hARD1ΔN

**Figure 7:**
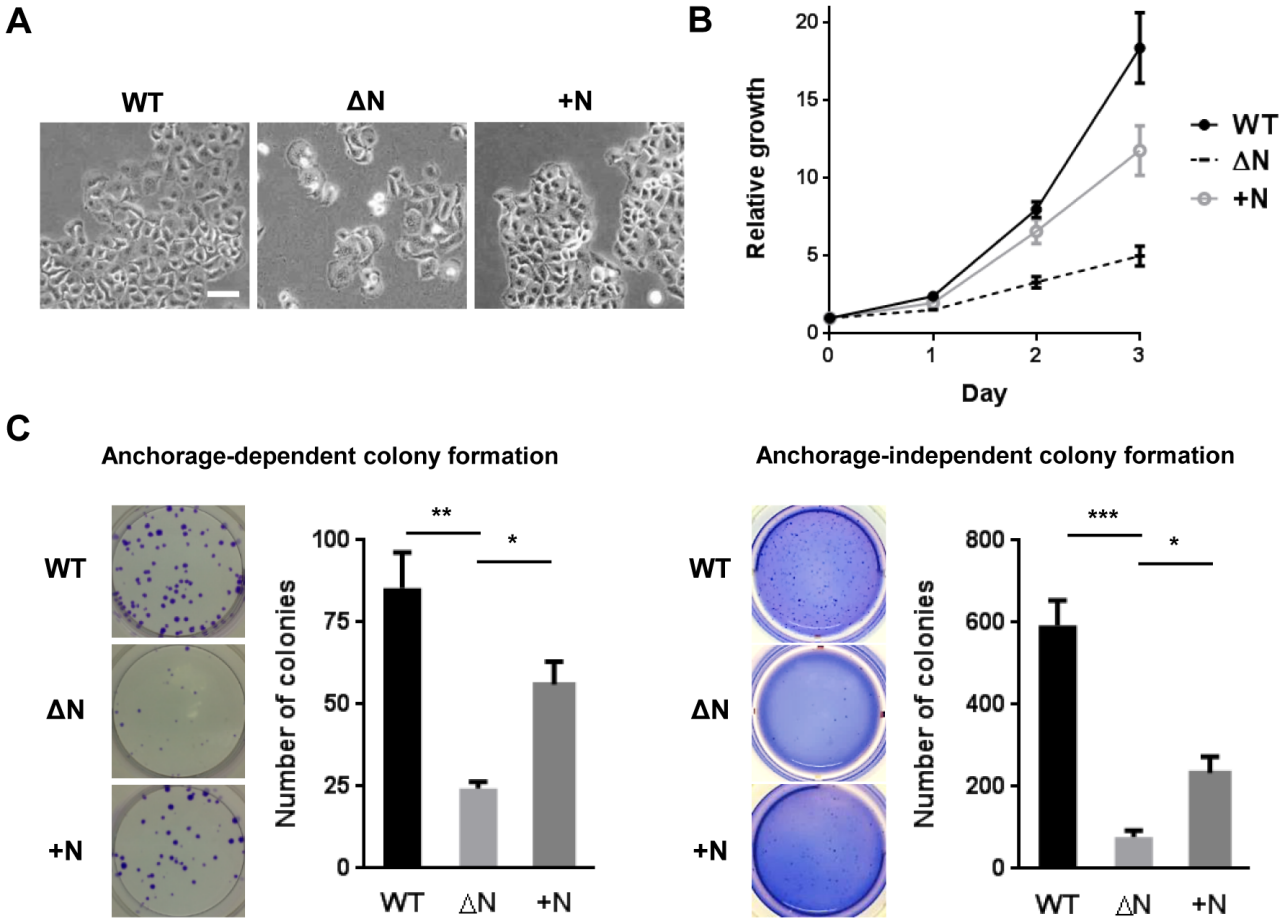
NLS insertion into hARD1 rescues impaired cell growth. **A**. The insertion of exogenous NLS into hARD1 restored the morphological alterations of ΔN-expressing cells. HeLa cells stably expressing hARD1+N were established, and cell morphology was observed under phase-contrast microscope. Scale bar, 50 µm. **B** and **C**. The exogenous NLS partially recovered the impaired growth of cells expressing the NLS deletion mutant. B, cell growth of hARD1+N stable cells with WT and ΔN stable cells was monitored by MTS assay. The results of each group were normalized to day 0. C, HeLa cells stably expressing hARD1 WT, ΔN, and +N were subjected to anchorage-dependent (left) and -independent (right) colony formation assays.

We next focused on whether restoration of nuclear localization of hARD1ΔN could also rescue alterations in cell morphology and proliferation. To this end, HeLa cells were stably transfected with GFP-hARD1+N (Fig. 5D) and observed under phase-contrast microscopy. Consistent with restoration of the cell cycle, the irregular hARD1ΔN cells recovered the normal shape of HeLa cells after ectopic insertion of the NLS (Fig. 7A).

Cell proliferation measured by MTS and anchorage-dependent/independent colony formation assays also showed partial recovery in hARD1+N cells (Fig. 7B, C), indicating that impaired nuclear localization was the major causative factor for decreased cell growth in hARD1ΔN cells. These results were also verified in A549 cells (Fig. S2).

## Discussion

Because the cell cycle alterations caused by functional changes to cell cycle regulatory proteins are tightly correlated with the uncontrolled proliferation of cancer cells, modulation of the cell cycle in proliferating cells is a promising approach for anticancer therapy. In this study, we observed that hARD1 translocates to the nucleus of HeLa cells during the S phase (Fig. 1) and inhibition of nuclear translocation caused alterations in cell cycle, morphology, and consequently, cell proliferation (Fig. 3, 4). These phenomena were reversed when nuclear localization was restored by NLS insertion (Fig. 5, 6, 7). We also have demonstrated that a putative NLS, which has a debatable effectiveness because of a low NLS score and ambiguous cellular localization of ARD1 in different studies, is capable of mediating nuclear import of hARD1 (Fig. 2).

As its name suggests, ARD1 is reported to play roles in cell proliferation and cancer in yeast and mammalian cells. This implies involvement of ARD1 in the cell cycle, explaining the high homology and wide distribution of ARD1 throughout its broad range of species and tissues. Here, we propose that hARD1 nuclear translocation plays a role in cell cycle progression.

Several studies on the subcellular localization of ARD1 have had controversial results, showing it in various locations in a variety of cell lines and by different isoforms of ARD1 [18]–[20]. Some reports have described opposing locations of hARD1 in different cell lines, suggesting that localization is dependent on the cell context. Like the location, the role of ARD1 in cancer, whether a positive or negative correlation, remains controversial. These variable properties of ARD1 imply the presence of other relevant factors involved in its activity.

Our study's finding that the function of hARD1 can be influenced by nuclear translocation suggests a possible explanation for the inconsistent and variable behaviors of hARD1. Another possibility can be autoacetylation of hARD1, which contributes to its functional activation in cell proliferation [22]. In addition, our group previously suggested that ARD1 variants in mouse and human cells may serve different roles [4]. Elucidating the correlation of these factors with cancer should be addressed and needs to be considered in dealing with ARD1 activity.

The subcellular localization of a protein is highly correlated to its biological function and is important in cell cycle progression. This aspect enables the rapid control of a protein's function and is quite suitable for the regulation of dynamic cell cycle transitions. Because the basal level of nuclear hARD1 is a relatively small fraction of the total expression level, the protein may act primarily in the cytoplasm, where it is more abundant. For example, N-terminal acetylation during protein synthesis, a well-known function of hARD1, occurs cooperatively with NATH in the cytoplasm [18]. After it is imported to the nucleus, however, hARD1 might serve the novel function of contributing to cell cycle progression, which means the subcellular localization imparts diverse functionality to hARD1.

Since hARD1 is imported to the nucleus during the S phase, immediately after cyclin E induction, its translocation has a presumptive role in S phase progression or the subsequent G2/M transition. Flow cytometry analysis showed that cells expressing a NLS-deleted mutant arrested in the G2/M phase (Fig. 4), with morphology suggesting that the cells were stuck in mitosis (rounded shape) or underwent mitotic slippage (enlarged and multinucleated) (Fig. 3), indicating an inability for the cells to properly complete mitosis.

To assess the effects of nuclear translocation independently, we inserted an exogenous NLS into the NLS deletion mutant and observed whether it could rescue the mutant phenotype. The observed recovery of localization, cell cycle, and cellular growth indicated that a change in location of hARD1 alone is enough to cause alterations in cells. Although the cell shape and cycle were mostly recovered by insertion of the exogenous NLS, cell growth showed only a partial recovery of approximately 40–70% of WT (Fig. 7). One possible explanation for this is the partial restoration of nuclear localization by the exogenous NLS (Fig. 5C, D).

All of these findings suggest the importance of nuclear hARD1 in the cell cycle, although the precise physiological role of imported hARD1 and its underlying mechanisms still need further investigations. In addition, investigating subcellular localization of hARD1 in different kinds of tumors or under distinct stimuli may advance our understanding of the physiological significance of hARD1 translocation.

Here, we revealed that the translocation of hARD1 into the nucleus contributes to cell cycle progression and proliferation. This study is the first report to describe the significance of subcellular localization of ARD1 with its function and provides one possible explanation for previous complicated and conflicting results on its role in cancer. In addition, this study adds a novel regulatory mechanism in cell cycle progression, mediated by an evolutionarily conserved protein, ARD1.

## Materials and Methods

### Cell culture and synchronization

HeLa, A549 and HEK293T cells were grown in DMEM supplemented with 10% fetal bovine serum (FBS) and 1% penicillin/streptomycin in 5% CO_2_ humidified atmosphere at 37°C. For serum starvation, HeLa cells were incubated in DMEM without serum for 48 h and re-stimulated with DMEM containing 10% FBS. A double thymidine block was performed to synchronize cell populations in S phase. HeLa cells were treated with 2 mM thymidine (Sigma-Aldrich) for 18 h, cultured for 9 h in normal growth medium, and then treated with thymidine for another 16 h. After removing the thymidine, the cells were released to normal medium and harvested at the indicated times.

### Generation of hARD1 constructs

To generate hARD1 expression vectors, hARD1 cDNA was amplified by PCR and subcloned into a pEGFP-C3 vector (GFP-hARD1 WT). Deletion and insertion of NLS (GFP-hARD1ΔN and GFP-hARD+N, respectively) were performed using the Muta-Direct Site Directed Mutagenesis kit (Intron) according to the manufacturer's instructions.

### Transfection and establishment of stable cell lines

Transient and stable transfection were implemented using PolyFect reagent (Qiagen). For the establishment of stable cells, hARD1 wild type and mutant plasmids were transfected in HeLa and A549 cells and maintained in complete DMEM with G418 (600 µg/mL). After several days, the surviving colonies were selected and amplified.

### Fluorescence microscopy

For the analysis of hARD1 localization by flourescence microscopy, HeLa cells were seeded onto the glass coverslips in 24-well plates. After synchronization by double thymidine block and release, cells were fixed in 4% PFA for 20 min and permeabilized in 0.3% triton X-100 in PBS 5 min at room temperature. Then, cells were incubated with hARD1 antibody (Santa Cruz) and visualized with Alexa 488-conjugated igG (Molecular Probes). Nucleus staining was performed with Hoechst 33342 (Molecular Probes). The immunofluorescence was visualized using an Axiovert M200 microscope (Carl Zeiss).

### Protein extraction, fractionation, and western blotting

Cells were lysed in lysis buffer (20 mM Tris-HCl [pH 7.5], 150 mM NaCl, 1 mM Na_2_EDTA, 1 mM EGTA, 1% triton X-100, 2.5 mM sodium pyrophosphate, 1 mM beta-glycerophosphate, and 1 mM Na_3_VO_4_). Lysates were immunoblotted with the corresponding primary antibodies. Antibodies for hARD1, cyclin B1, cyclin E, p53, and green fluorescent protein (GFP) were purchased from Santa Cruz Biotechnology, and Aurora kinase A (AURKA) and Aurora kinase B (AURKB) were from Cell Signaling.

For the nuclear/cytosolic fractionation, cultured cells were washed with PBS, homogenized in buffer A (10 mM HEPES (pH 7.4), 1.5 mM MgCl_2_, 10 mM KCl, 0.5 mM DTT, 0.1% NP-40), and centrifuged for 10 min at 600×*g* in 4°C. For the cytosolic fraction, supernatants were re-centrifuged for 30 min at 15,000 rpm. For nuclear extracts, the nuclear pellet was washed with buffer A, resuspended in buffer C (10 mM HEPES (pH 7.4), 1.5 mM MgCl_2_, 0.5 mM DTT, 20% glycerol, 0.5 mM PMSF, 0.2 mM EDTA, and 420 mM NaCl), centrifuged for 30 min at 15,000 rpm, and the supernatant isolated.

### Flow cytometry analysis

For the flow cytometry assay, cells were collected, fixed in 70% ethanol, and stored at −20°C. Cells were then washed, resuspended in PBS containing 1 mg/mL RNase A, and incubated at 37°C for 30 min; this was followed by propidium iodide (PI, 30 µg/mL) staining for 15 min. The DNA content of cells was assessed with a FACS-CALIBUR system (BD Biosciences), and cell cycle profiles were analyzed with the BD FACSuite software. At least 30,000 cells in each sample were analyzed to obtain a measurable signal, using the same instrument setting.

### Cell proliferation assay

The proliferation rates were measured using a Cell Proliferation Assay kit (Promega) according to the manufacturer's instructions. Briefly, 3×10^3^ cells/well were seeded on 96-well plates and allowed to grow. After the indicated time, 20 µL of substrate solution was added and the cells were incubated for 1 h to allow color development. The absorbance at 492 nm was measured to indicate the number of proliferating cells.

### Colony formation assay

Colony formation assays were performed as described previously [22]. For anchorage dependent colony formation, stable cells were trypsinized, counted, and seeded at a density of 100 cells/well in 6-well plates. After 2 weeks, the cells were fixed and stained with 0.005% crystal violet and colonies were counted. For anchorage independent colony formation, cells were trypsinized, counted, and resuspended in 0.5 mL DMEM containing 0.4% agar. The cell mixture was seeded on a layer of 1% agar in 12-well plates and allowed to grow. After 2 weeks of incubation, colonies were stained with 0.005% crystal violet and counted.

### Statistical analysis

Results are presented as means ± S.D. *P* values were calculated by applying the unpaired two-tailed Student's *t* test. The difference was considered statistically significant when *P*<0.05.

## Supporting Information

Figure S1 **Serum stimulation increased levels of nuclear hARD1.** Serum-starved HeLa cells were re-stimulated with 10% FBS for the indicated times. Nuclear and cytosolic proteins were fractionated and immunoblotted with anti-hARD1, cyclin E, Histone H1, and α-Tubulin antibodies.
(TIF)

Figure S2 **Nuclear hARD1 is essential for proper growth of A549 cells.** **A**. Constitutive expression of the hARD1 ΔN leads to morphological changes that are restored by insertion of exogenous NLS. A549 cells stably expressing hARD1 WT, ΔN, and +N were established, and cell morphology was examined. Scale bar, 50 µm. **B**. hARD1ΔN-expressing cells showed a decrease in cell growth, and this was restored in cells expressing hARD1+N cells. Cell growth of A549 stable cell lines was assessed using an MTS assay. **C**. hARD1ΔN-expressing cells showed moderate G2/M arrest, which was rescued by hARD1+N expression.
(TIF)
