# Supplementary figures and images for: Nuclear Translocation of hARD1 Contributes to Proper Cell Cycle Progression
Source: PLoS One. 2014 Aug 18;9(8):e105185. doi: 10.1371/journal.pone.0105185 (PMC4136855; doi:10.1371/journal.pone.0105185)

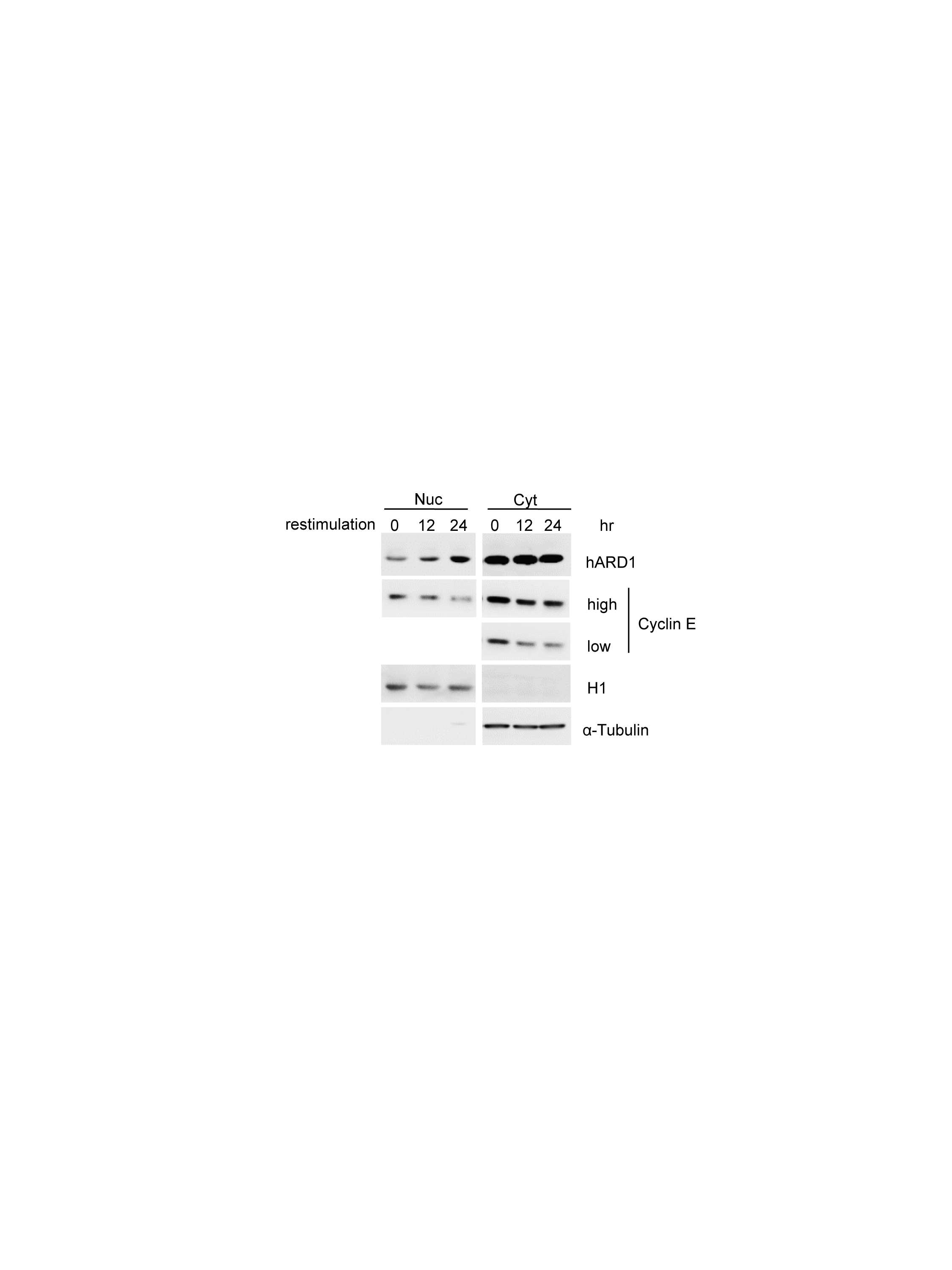

Supplement: Figure S1 — Serum stimulation increased levels of nuclear hARD1. Serum-starved HeLa cells were re-stimulated with 10% FBS for the indicated times. Nuclear and cytosolic proteins were fractionated and immunoblotted with anti-hARD1, cyclin E, Histone H1, and α-Tubulin antibodies. (TIF) [file pone.0105185.s001.tif]

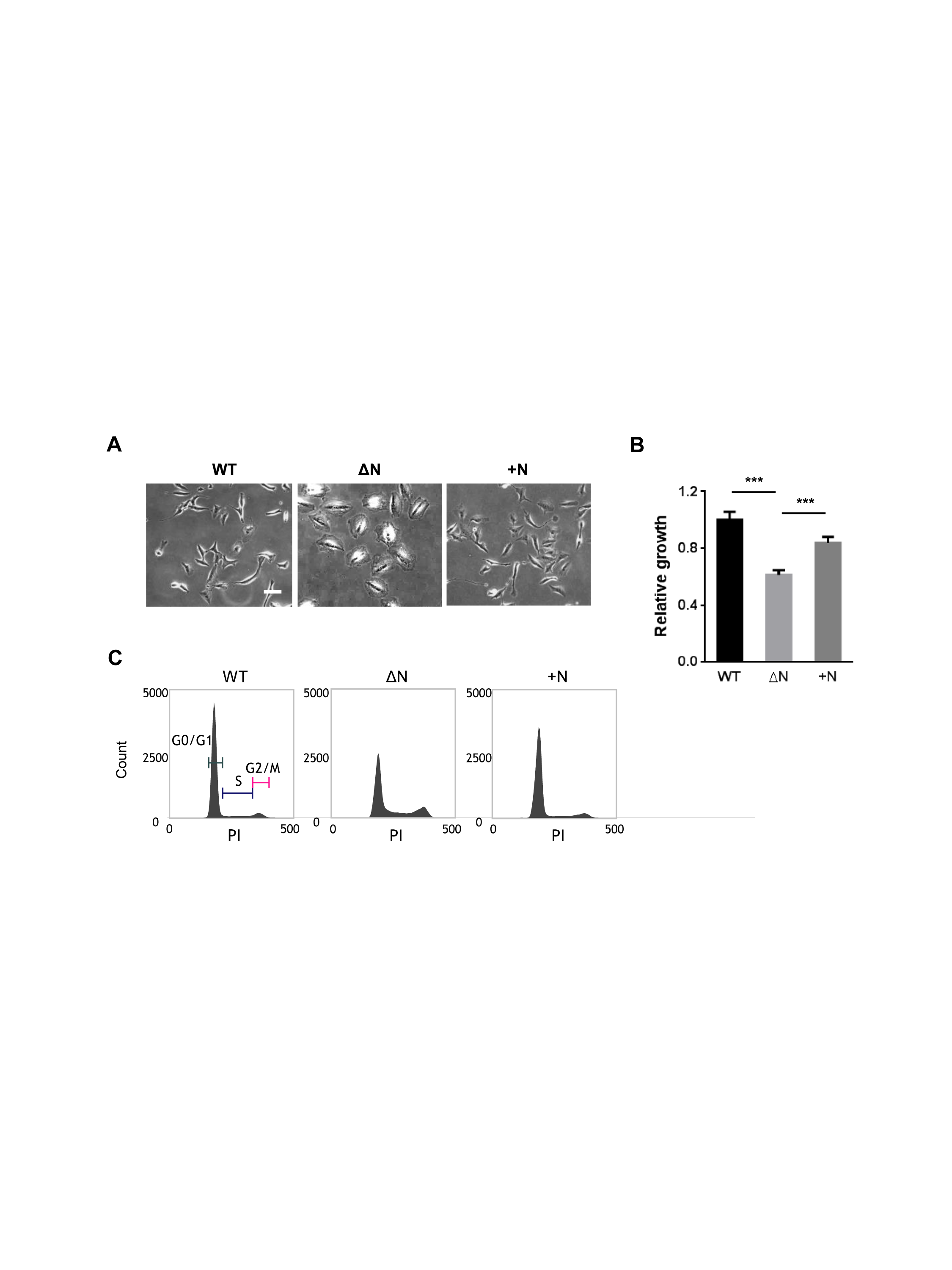

Supplement: Figure S2 — Nuclear hARD1 is essential for proper growth of A549 cells. A. Constitutive expression of the hARD1 ΔN leads to morphological changes that are restored by insertion of exogenous NLS. A549 cells stably expressing hARD1 WT, ΔN, and +N were established, and cell morphology was examined. Scale bar, 50 µm. B. hARD1ΔN-expressing cells showed a decrease in cell growth, and this was restored in cells expressing hARD1+N cells. Cell growth of A549 stable cell lines was assessed using an MTS assay. C. hARD1ΔN-expressing cells showed moderate G2/M arrest, which was rescued by hARD1+N expression. (TIF) [file pone.0105185.s002.tif]
